# Splenectomy is associated with an aggressive tumor growth pattern and altered host immunity in an orthotopic syngeneic murine pancreatic cancer model

**DOI:** 10.18632/oncotarget.21331

**Published:** 2017-09-28

**Authors:** Ho Kyoung Hwang, Takashi Murakami, Tasuku Kiyuna, Se Hoon Kim, Sung Hwan Lee, Chang Moo Kang, Robert M. Hoffman, Michael Bouvet

**Affiliations:** ^1^ Department of Surgery, University of California, San Diego, CA, USA; ^2^ AntiCancer, Inc., San Diego, CA, USA; ^3^ Department of Surgery, Division of Hepatobiliary and Pancreas Surgery, Severance Hospital, The Graduate School, Yonsei University College of Medicine, Seoul, Korea; ^4^ Graduate School of Medicine, Yokohama City University, Yokohama, Japan; ^5^ Department of Orthopedic Surgery, University of the Ryukyus, Okinawa, Japan; ^6^ Department of Pathology, Severance Hospital, Yonsei University College of Medicine, Seoul, Korea

**Keywords:** pancreatic cancer, orthotopic mouse models, splenectomy, tumor infiltrating lymphocytes, metastases

## Abstract

The purpose of this study was to investigate whether splenectomy influences the tumor growth and metastatic pattern in an orthotopic syngeneic murine pancreatic cancer model. Murine pancreatic cancer cells (PAN02) were subcutaneously injected into the flanks of nude mice. A small tumor fragment (3 mm^2^), harvested from a subcutaneous tumor. was orthotopically implanted in the tail of the pancreas of C57/BL6 mice without splenectomy (control group, n=15) or with simultaneous splenectomy (splenectomy group, n=15). Tumor growth and metastatic patterns were analyzed by laparotomy at 21 days after surgery. No tumor growth was found in 5 mice (33.3%) of the control group and 1 mouse (6.7%) of the splenectomy group (*p*=0.169). Tumor volume was significantly larger in splenectomy group *(p*=0.013). Peritoneal seeding was more frequently observed in the splenectomy group (11 (73.3%) *vs.* 4 (26.7%), *p*=0.011). There were no differences in the number of liver and kidney metastasis between the two groups. The ratios of tumor-infiltrating CD4^+^ to FoxP3^+^ and CD8^+^ to FoxP3^+^ were significantly higher in the control group compared to the splenectomy group (8.2 ± 9.3 vs. 2.4 ± 1.5, *p*=0.046; 2.5 ± 1.4 vs. 1.5 ± 0.4, *p*=0.031, respectively). Splenectomy enhanced tumor growth and peritoneal seeding in an orthotopic syngeneic murine pancreatic cancer mouse model. The ramification of these results are discussed for pancreatic cancer treatment.

## INTRODUCTION

Distal pancreatectomy including splenectomy has been accepted as the standard procedure for left-sided pancreatic ductal adenocarcinoma (PDAC) [1, 2]. Splenectomy can clear regional lymph nodes of pancreatic cancer cells. If the tumor is located away from the splenic hilum and there is no evidence of nodal metastasis around the spleen, splenectomy may not be necessary for distal pancreatectomy. We previously reported in an international multi-center survey, that in such cases, splenectomy-omitting radical distal pancreatectomy would be feasible [3].

Splenectomy has been shown to have a negative effect on cancer survival of patients with gastric and colon cancer [4–6]. Schwarz et al reported that splenectomy had a negative influence on long-term survival after pancreatectomy for PDAC [7]. The number of hepatic and lung metastases increased in splenectomized mice using colon cancer and liver tumor models [8–10]. In terms of PDAC, there is no mouse model study to demonstrate the role of the spleen for tumor growth and metastasis or oncologic outcome.

The purpose of this study was to investigate whether splenectomy influences the tumor growth pattern, as well as host immunity, in an orthotopic syngeneic murine pancreatic cancer model.

## RESULTS

### Tumor growth patterns

**Figure 1:**
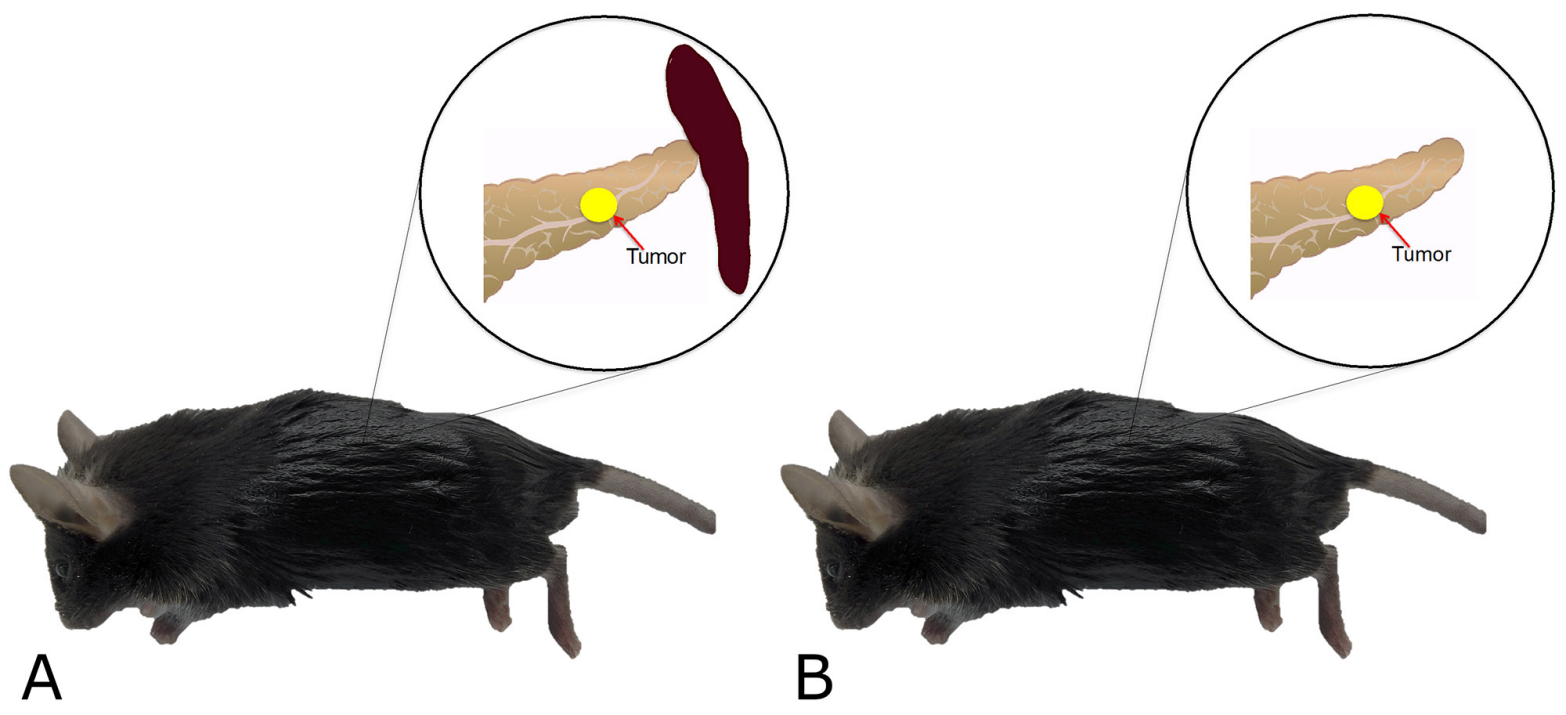
Schema of experimental plan Small fragments (3 mm) of murine pancreatic cancer (PAN02), previously grown subcutaneously, were orthotopically implanted in the pancreatic tail of C57/BL6 mice without splenectomy (**A**, control group) or with splenectomy (**B**, splenectomy group).

**Figure 2:**
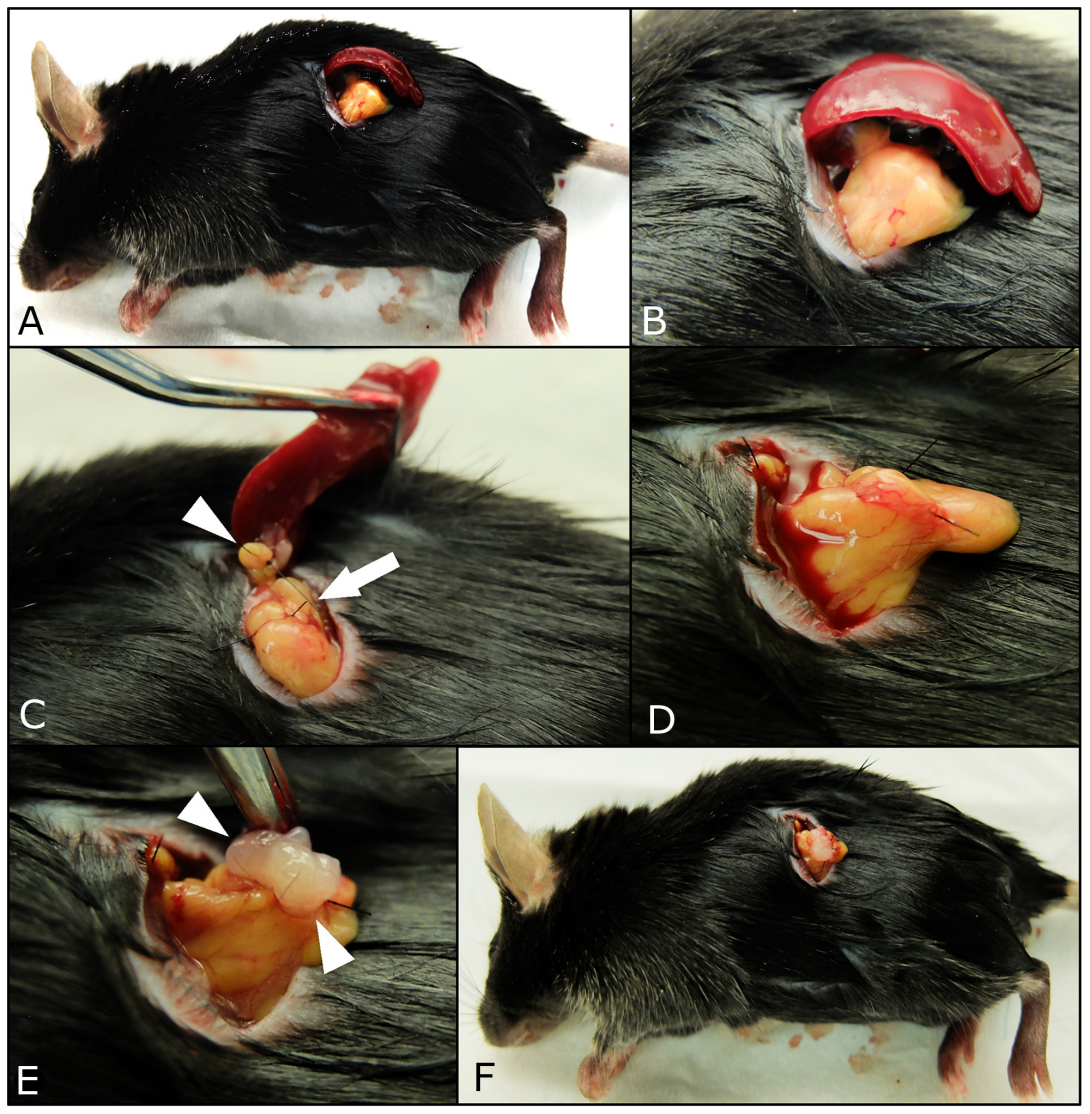
Procedures of surgical orthotopic implantation (SOI) of pancreatic cancer tumor fragments with simultaneous splenectomy A small 6-10 mm transverse incision was made on the left flank of mice through the skin and peritoneum. The pancreatic tail and spleen were exposed through this incision **(A, B)**. The splenic artery and vein in the splenic hilum (white arrows) and short gastric vessels communicating with the splenic upper pole (white arrow head) were securely ligated **(C)**. After the spleen was removed **(D)**, a single tumor fragment (3 mm^3^) was sutured to the pancreas tail using 7-0 nylon surgical sutures (white arrow head), (**E**). Upon completion, the pancreatic tail was returned to the abdomen, and the incision was closed in one layer using 6-0 nylon surgical sutures (**F**).

**Figure 3:**
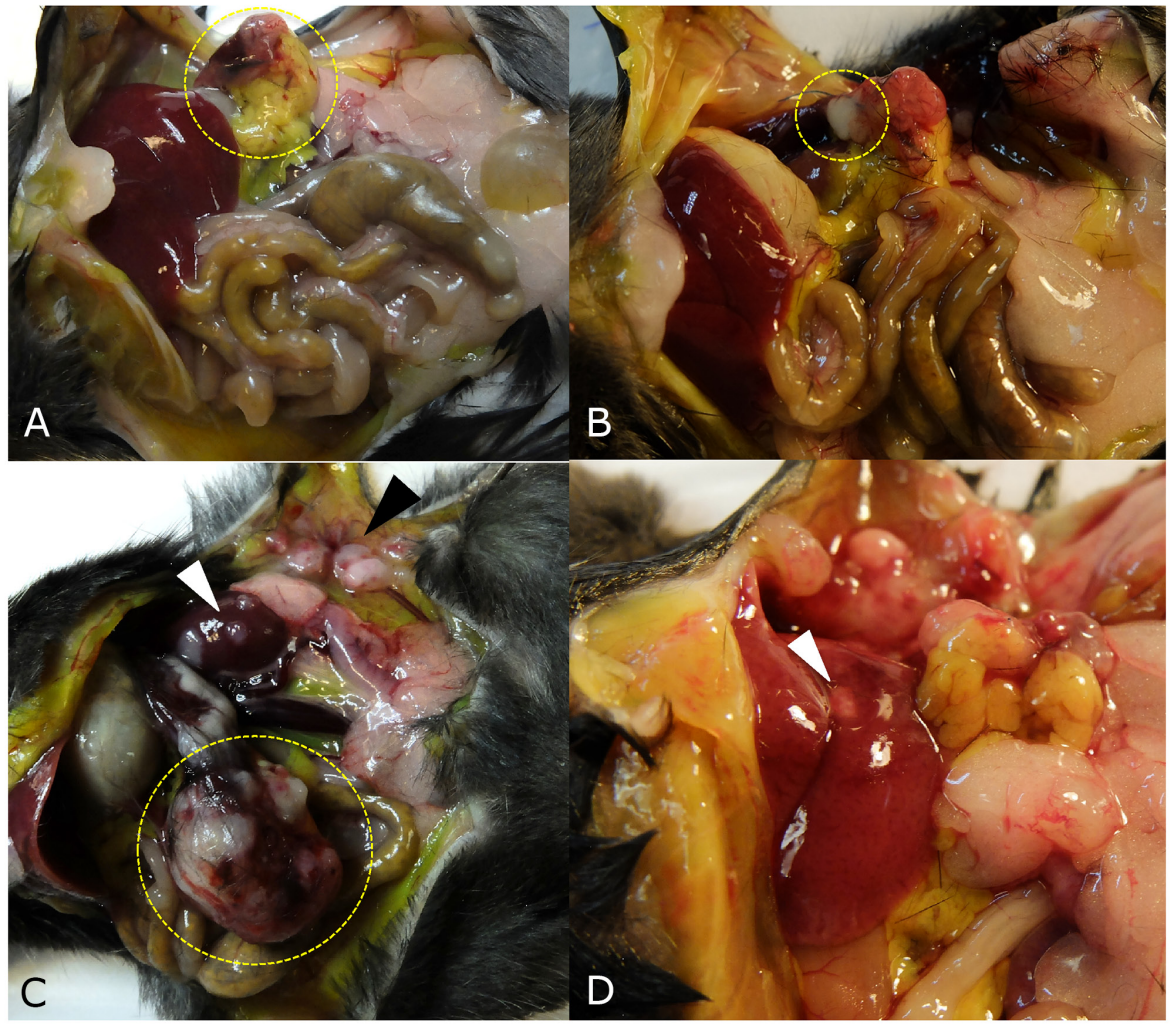
Tumor growth patterns at laparotomy **(A)** The implanted tumor disappeared and only peritoneal fat was attached around surgical bed in one mouse (yellow dotted circle). **(B)** If the tumor grew to less than 5 mm, the tumor growth pattern was defined as ‘no growth’ (dotted circle) in a mouse in the control group. **(C)** Large pancreatic tumor (yellow dotted circle), kidney metastasis (white arrow head), and peritoneal seeding (black arrow head) were observed in a mouse in the splenectomy group. **(D)** Liver metastasis was observed in one mouse (white arrow head).

**Figure 4:**
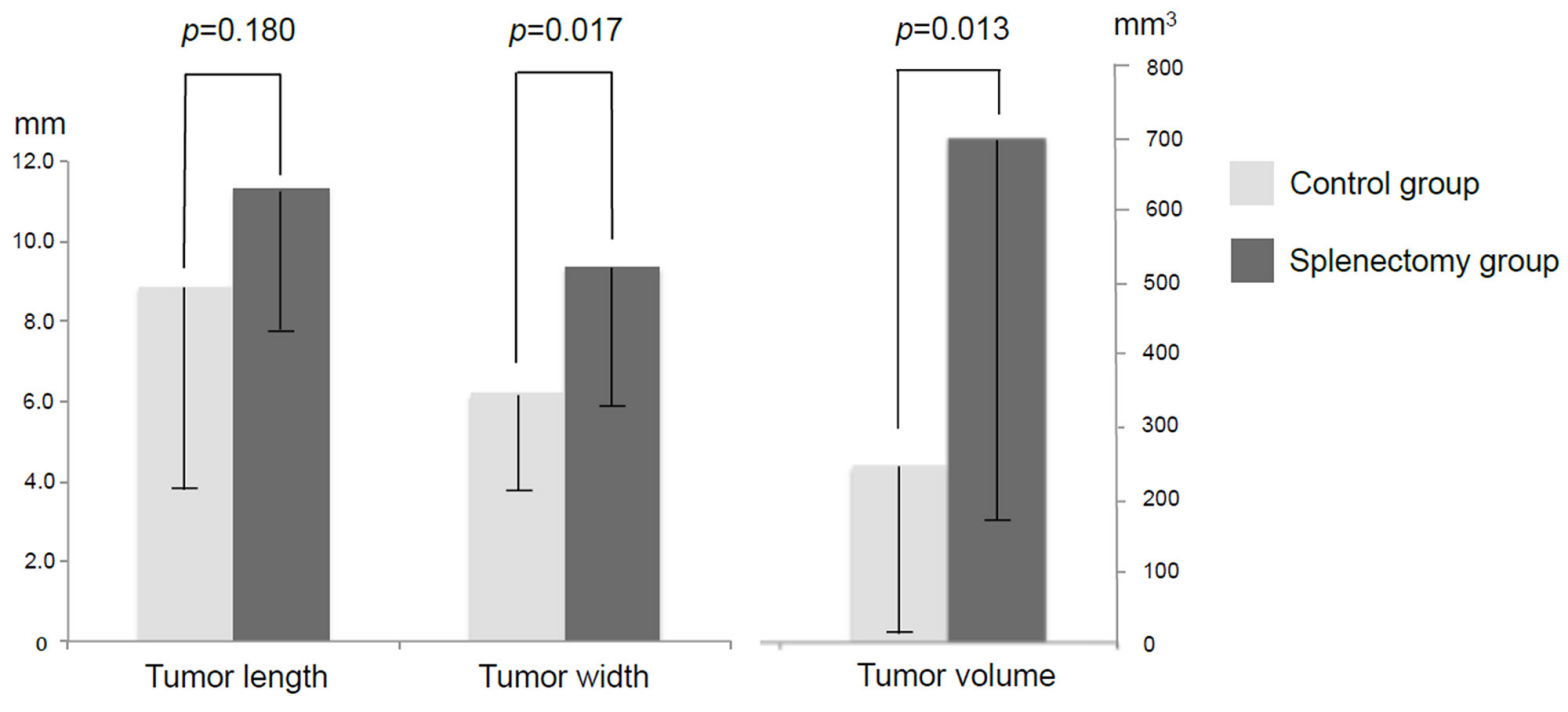
Tumor size and volume in splenectomy and control groups Tumor width and total tumor volume were significantly larger in the splenectomy group compared to the control group.

The mice were divided into two groups according to splenectomy or no splenectomy (Figure 1). After the spleen was removed, a single tumor fragment was sutured to the pancreatic tail by surgical orthotopic implantation (SOI) (Figure 2). Fifteen mice were used in both the control and splenectomy groups. Body weight at SOI and laparotomy was not different between the splenectomy and non-splenectomy groups. In the splenectomy group, one mouse was died at 19 days after surgery. The mouse had severe ascites and multiple peritoneal seeding at laparotomy. In one mouse in the control group, the implanted tumor could not be identified at the pancreatic tail and only suture material was observed. Peritoneal fat was attached around the surgical bed. We defined this mouse as ‘tumor rejection’. If the implanted tumor grew to less than 5 mm, the mouse was defined as ‘no growth’. No tumor growth was observed in 5 mice (33.3%) of the control group and 1 mouse (6.7%) of the splenectomy group. There was no statistical difference in these parameters (Table 1) (Figure 3). Tumor volume was significantly larger in splenectomy group *(p*=0.013) (Table 1) (Figure 4).

**Table 1:** Tumor growth and metastatic pattern in splenectomized and control mice

	Control group (n=15)	Splenectomy group (n=15)	*p*-value
Body weight at SOI	19.3 ± 2.6	18.18 ± 3.2	0.320
Body weight at laparotomy	21.8 ± 2.6	21.5 ± 3.4	0.797
Postoperative death			1.000
No	15 (100%)	14 (93.3%)	
Yes	0	1 (6.7%)	
Tumor rejection			1.000
No	14 (93.3%)	15 (100%)	
Yes	1 (6.7%)	0	
Tumor growth			0.169
No	5 (33.3%)	1 (6.7%)	
Yes	10 (66.7%)	14 (93.3%)	
Tumor size (length), mm	8.8 ± 5.0	11.3 ± 4.3	0.180
Tumor size (width), mm	6.2 ± 2.7	9.3 ± 3.8	0.017
Tumor volume, mm^3^	244.1 ± 239.5	697.9 ± 543.4	0.013
Ascites			0.068
No	10 (66.7%)	5 (33.3%)	
Yes	5 (33.3%)	10 (66.7%)	
Peritoneal seeding			0.011
No	11 (73.3%)	4 (26.7%)	
Yes	4 (26.7%)	11 (73.3%)	
Liver metastasis			1.000
No	14 (93.3%)	15 (100%)	
Yes	1 (6.7%)	0	
Kidney metastasis			1.000
No	14 (93.3%)	14 (93.3%)	
Yes	1 (6.7%)	1 (6.7%)	

### Tumor metastases patterns

Severe ascites was observed in 5 (33.3%) mice of the control group and 10 (66.7%) mice of the splenectomy group but without statistical significance (*p*=0.068). Peritoneal seeding was more frequently identified in the splenectomy group when compared to the control group (11 [73.3%] *vs.* 4 [26.7%], respectively, *p*=0.011). Liver metastasis was observed in only one mouse of the control group. Kidney metastases were observed in one mouse of each group (Table 1).

### Tumor-infiltrating T lymphocyte analysis

The ratios of CD4^+^ to FoxP3^+^ and CD8^+^ to FoxP3^+^ were significantly higher in control group compared to splenectomy group (8.2 ± 9.3 vs. 2.4 ± 1.5, *p*=0.046; 2.5 ± 1.4 vs. 1.5 ± 0.4, *p*=0.031, respectively) (Table 2).

**Table 2:** Tumor infiltrating lymphocyte subset ratios in the splenectomized and control mice

	Control group (n=14)	Splenectomy group (n=15)	*p*-value
CD4^+^/FoxP3^+^	8.2 ± 9.3	2.4 ± 1.5	0.046
CD8^+^/FoxP3^+^	2.5 ± 1.4	1.5 ± 0.4	0.031

## DISCUSSION

This study investigated the impact of splenectomy on tumor growth and host immunity in an orthotopic syngeneic pancreatic cancer mouse model. The results showed that the tumor volume was significantly larger and peritoneal seeding was more frequent in the splenectomy group compared to the control group. The tumor growth pattern in the splenectomized mice was thus associated with more aggressive behavior.

Although the impact of splenectomy on experimental models with several kinds of cancer has been studied, the exact mechanism of splenectomy on cancer growth remains uncertain. Shiratori et al [10] and Imai et al [9] reported that the number of hepatic and lung metastases increased in splenectomized mice using colon-cancer and liver-tumor models, respectively. They postulated that the mechanism of the increased liver and pulmonary metastases was due to decreased activity of NK cells after splenectomy [9, 10]. In these previous reports to demonstrate the impact of splenectomy on tumor growth, cancer cells were directly injected into the superior mesenteric vein or liver parenchyma to establish an experimental model of liver metastasis of colon cancer. When the cancer cells are injected in a vein or into the liver parenchyma, there is a chance of widespread cancer cell dissemination or intravasation into the portal vein or hepatic vein. With these experimental designs, cancer cells enter the circulation at the initial stage of tumor growth which is a different situation from natural tumor growth and metastasis. In our study, we orthotopically implanted tumor fragments in the pancreas tail which is a very similar situation to the clinical setting of patients with left-sided pancreatic cancer. In those cases, we can try to preserve the spleen in well-selected patients (for example, no evidence of tumor invasion to the spleen, no splenic hilum lymph node metastasis, small sized tumor etc.) during distal pancreatectomy.

Another possible mechanism for the splenectomy effect on aggressive tumor behavior is that regulatory T cells may increase in some organs. Higashijima et al reported that the number of hepatic metastases significantly increased in the splenectomy group compared to the spleen-preserved group after colon cancer cells were injected into spleen [8]. They concluded that splenectomy enhanced hepatic metastasis through the increase of Foxp3 mRNA in the liver. Future studies will focus on the mechanism of increased metastases after splenectomy during pancreatectomy.

Our results showed that the relative ratios of CD4^+^ helper T lymphocyte to FoxP3^+^ Treg and CD8^+^ cytotoxic T lymphocytes to FoxP3^+^ Treg were significantly higher in the control group compared to the splenectomy group. We also previously reported that the ratios of helper T cells or cytotoxic T cells to Treg cells were significantly related to survival in patients with gastric and pancreatic cancer [11, 12]. Shang reported in their review that high FoxP3^+^ Treg infiltration was significantly associated with shorter overall survival in the majority of cases [13]. Future experiments will measure other immune cells including myeloid-derived suppressor cells, NK cells, M1 and M2 macrophages as well as time-course studies of the actual numbers of CD4^+^ helper lymphocytes, FoxP3^+^Treg, and CD8^+^ cytotoxic T lymphocytes during pancreatic tumor growth and metastasis in control and splenectomized mice.

When we consider the clinical setting, spleen-preserving distal pancreatectomy can be attempted at the time of cancer surgery in patients with PDAC. In addition, the impact of splenectomy on tumor recurrence and oncologic outcomes will be further investigated with orthotopic syngeneic models using other mouse pancreatic cancer cell lines as well as patient pancreatic cancer in orthotopic humanized mouse models. Future experiments will determine if there is a difference in tumor recurrence or survival depending on spleen preservation or not during tumor resection in an orthotopic pancreatic cancer mouse model. Reimplantation of spleen fragments after pancreatectomy will also be done in the mouse models to determine if this can restore efficient antitumor mouse function. Clinical studies will also be performed to further understand the tumor immunological effects of splenectomy which will be measured by flow cytometry as well as immunohistochemistry.

The present experiment, to our knowledge, is the first study to examine the effect of splenectomy on tumor growth and host immunity in a syngeneic orthotopic murine pancreatic cancer model. In conclusion, splenectomy enhanced tumor growth and peritoneal seeding. The high ratio of tumor-infiltrating helper T or cytotoxic T lymphocytes to Treg cells seems to have important anti-tumor immunity in the control group. The present results suggest that splenectomy be avoided during pancreatic resection for PDAC whenever possible.

## MATERIALS AND METHODS

### Cell culture

The murine pancreatic cancer cell line, PAN02, was maintained in Dulbecco’s Modified Eagle’s Medium (DMEM; Invitrogen, Melbourne, Australia) supplemented with 10% fetal bovine serum (FBS; Sigma-Aldrich, St. Louis, MO). The cells were incubated at 37°C in a humidified incubator of 5% CO_2_ in air. The cells were collected after trypsinization and stained with trypan blue (Sigma-Aldrich, St. Louis, MO). Only viable cells which excluded trypan blue were counted with a hemocytometer (Hausser Scientific, Horsham, PA).

### Animals

Athymic nu/nu nude and C57/BL6 immunocompetent mice (AntiCancer Inc., San Diego, CA), 4–6 weeks old, were used for subcutaneous cancer cell injection to obtain stock tumor and orthotopic tumor implant models, respectively. Mice were kept in a barrier facility under HEPA filtration and fed with autoclaved laboratory rodent diet. All mouse surgical procedures were performed with the animals anesthetized by intramuscular injection of a 0.02 ml solution of 50% ketamine, 38% xylazine, and 12% acepromazine maleate. The animals were sacrificed at day 21 after surgery for measuring tumor growth. All animal studies were conducted with an AntiCancer Institutional Animal Care and Use Committee (IACUC)-protocol specifically approved for this study and in accordance with the principles and procedures outlined in the National Institute of Health Guide for the Care and Use of Animals under Assurance Number A3873-1.

### Subcutaneous cancer cell injection

PAN02 cells were harvested by trypsinization and washed twice with phosphate buffered saline (PBS; Sigma Aldrich). PAN02 cells (2 x 10^6^) were injected subcutaneously into the right and left flanks of nude mice within 30 min of harvesting. The resulting subcutaneous tumors were harvested for subsequent surgical orthotopic implantation (SOI).

### Orthotopic tumor implantation and simultaneous splenectomy

Surgical orthotopic implantation (SOI) of tumor fragments was performed in C57/BL6 mice, as previously described [14–17]. The mice were divided into two groups according to splenectomy or no splenectomy. A small 6-10 mm transverse incision was made on the left flank of the mouse through the skin and peritoneum. The pancreas tail and spleen were exposed through this incision, and a single tumor fragment (3 mm^3^), harvested from a subcutaneous tumor grown on a nude mouse, was sutured to the tail of the pancreas using 7-0 nylon surgical sutures (Dermalon™, Covidien; Medtronic Inc., MN, USA). In the control group, the pancreatic tail and spleen were returned to the abdomen, and the incision was closed in one layer using 6-0 nylon surgical sutures (Dermalon™). In the splenectomy group, the splenic artery and vein in the splenic hilum and short gastric vessels communicating with the splenic upper pole were securely ligated with 7-0 nylon sutures (Dermalon™). After the spleen was removed, a single tumor fragment was sutured to the pancreatic tail (Figure 2). Fifteen mice were used in both the control and splenectomy groups.

### Tumor growth and metastatic pattern analysis

Tumor growth patterns were analyzed by laparotomy. The animals were sacrificed 21 days after surgery. Tumor length and width were measured with calipers and tumor volume was calculated by the following formula: tumor volume = (length x width^2^)/2. In addition, the peritoneal cavity was carefully assessed for the evidence of peritoneal, hepatic, or other site metastases.

### Immunohistochemical staining and quantification of tumor-infiltrating T lymphocyte subsets

Immunohistochemical (IHC) staining for tumor-infiltrating T lymphocyte subsets was performed as previously described [11, 12]. Briefly, paraffin-embedded tumor tissue sections at a thickness of 4-μm were deparaffinized in xylene and rehydrated in decreasing concentrations of ethanol. Antigen retrieval was performed in citrate buffer in a microwave oven. Endogenous peroxidase activity was blocked by incubating the tissue with 3% hydrogen peroxide in methanol for 5 min. The sections were incubated for 60 min at room temperature with primary monoclonal antibodies against a cluster of differentiation antigens (CD4 [Cat. No. ab183685, 1:100, Abcam, Cambridge, UK], CD8 [Cat. No. ab203035, 1:100, Abcam], and Foxp3 [Cat. No. ab20034, 1:100, Abcam]), which were used to identify helper T lymphocytes, cytotoxic T lymphocytes, and regulatory T lymphocyte (Treg), respectively. After washing the sections twice with 0.05 mol/l Tris-buffered saline with 0.2% Tween-20, the sections were incubated with horseradish peroxidase-conjugated secondary antibody (Dako EnVision^®^Detection system, Dako, Glostrup, Denmark), followed by development with diaminobenzidine and counterstaining with hematoxylin. IHC staining was quantified by an experienced pathologist (SHK) who was blinded to the data. Three intense foci of staining in the tumor sections were selected and three high-power fields (magnification, x400) from each slide were selected for calculation of IHC staining result.

### Statistical analysis

All statistical analyses were performed with SPSS 20.0 software (IBM, New York, NY, USA). Categorical variables were compared using χ^2^ or Fisher exact tests. Continuous variables are presented as mean ± standard deviation (SD) and the significance was determined using Student’s *t*-test. Bar graph expressed mean value, and error bars show -SD. A *p*-value of 0.05 or less indicated statistical significance.
